# Supplementary figures and images for: A specimen of Rhamphorhynchus with soft tissue preservation, stomach contents and a putative coprolite
Source: PeerJ. 2015 Aug 20;3:e1191. doi: 10.7717/peerj.1191 (PMC4548500; doi:10.7717/peerj.1191)

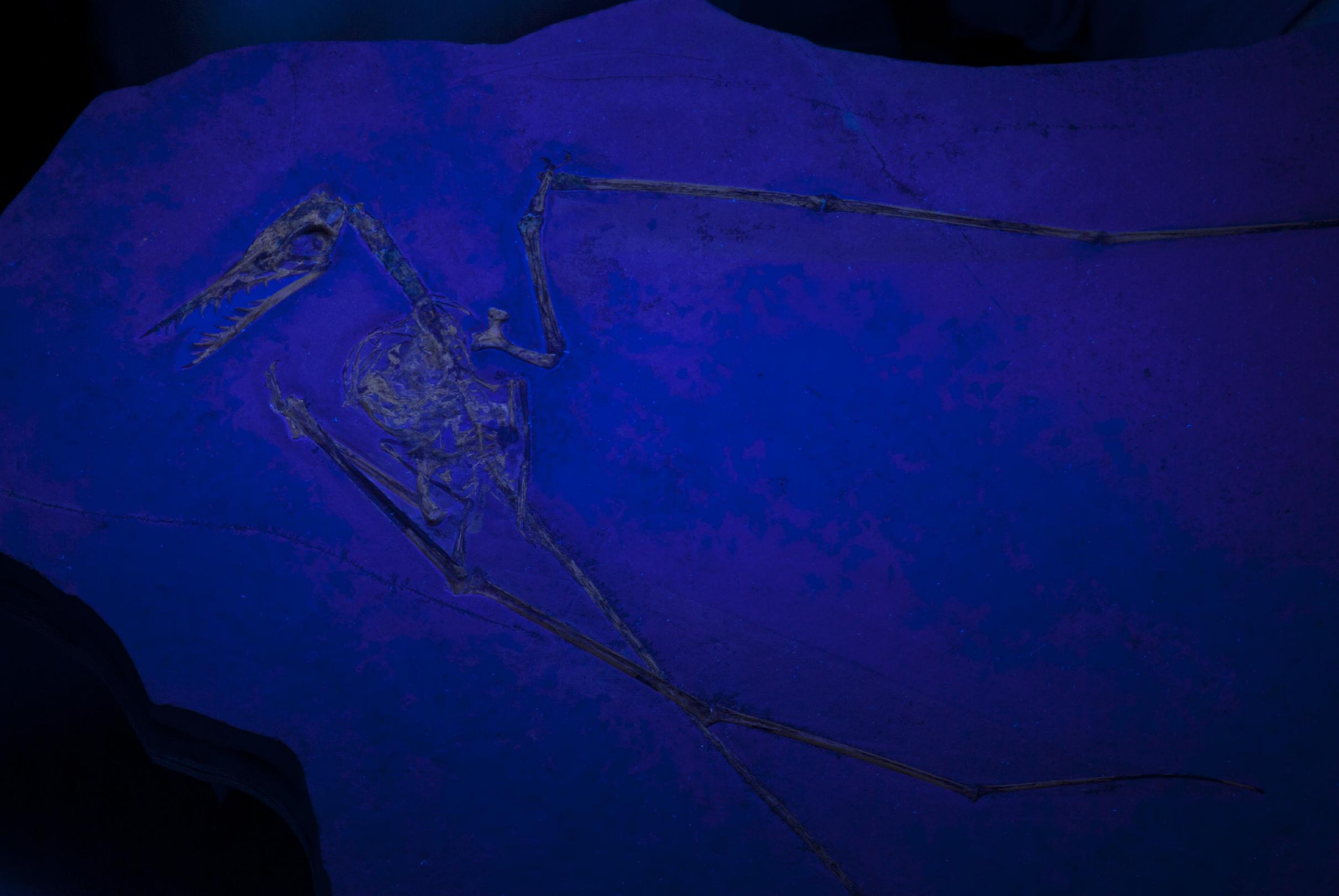

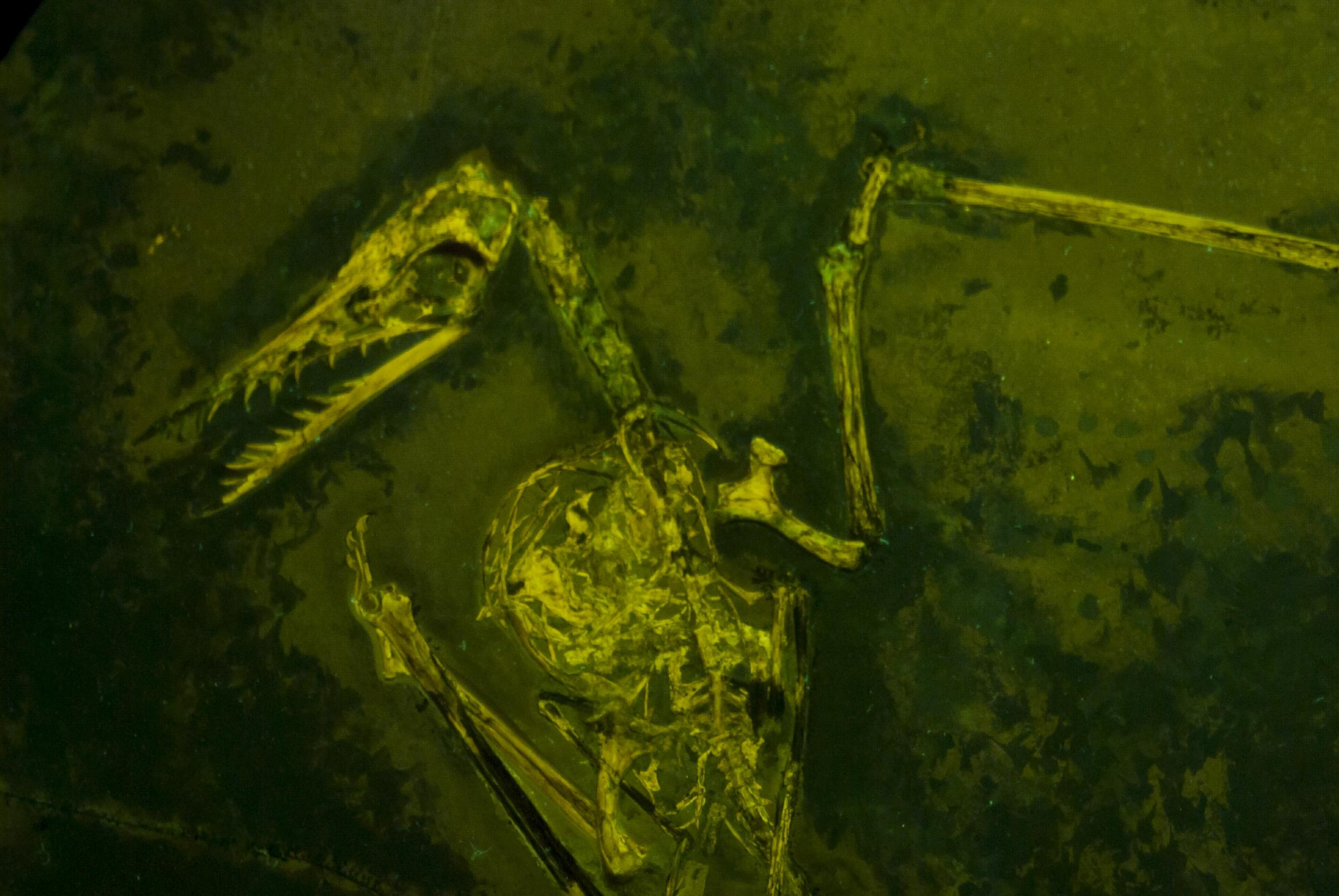

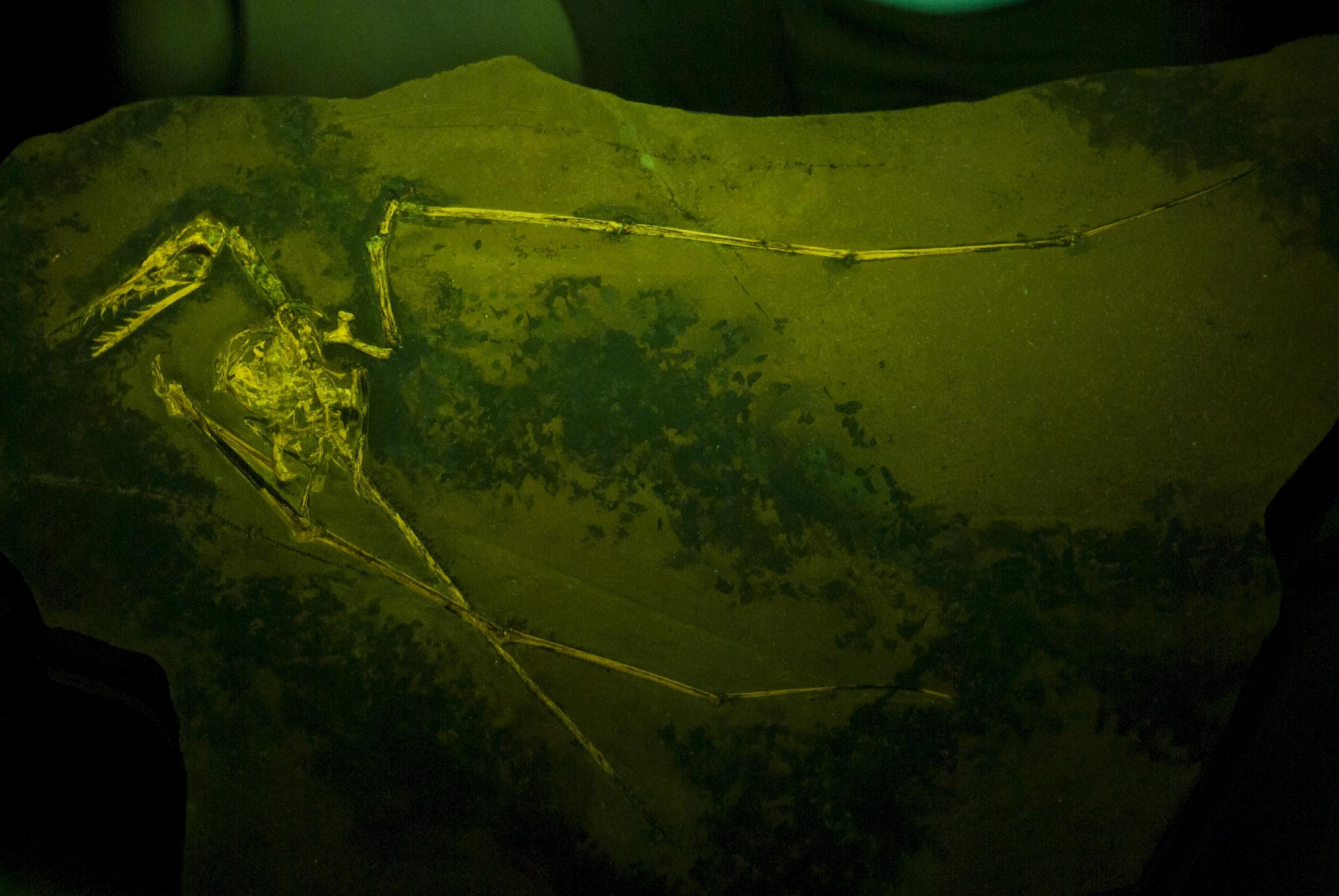

Supplement: Supplemental Information 1 [file peerj-03-1191-s001.pdf]
